# Correction: The importance of using a multi-dimensional scale to capture the various impacts of precarious employment on health: Results from a national survey of Chilean workers

**DOI:** 10.1371/journal.pone.0247613

**Published:** 2021-02-19

**Authors:** Alejandra Vives, Tarik Benmarhnia, Francisca González, Joan Benach

The following information is missing from the Funding statement: JB received financial support from ICREA under the ICREA Academia program.
