# Supplementary material for: The importance of using a multi-dimensional scale to capture the various impacts of precarious employment on health: Results from a national survey of Chilean workers
Source: PLoS One. 2020 Sep 24;15(9):e0238401. doi: 10.1371/journal.pone.0238401 (PMC7514101; doi:10.1371/journal.pone.0238401)
Supplement: S1 Table — (DOCX) [file pone.0238401.s001.docx]

Table S1: Interaction terms (95% C.I.) for the associations between study exposures and outcomes by sex, age, education and occupation.

|  |  | General Health | | | Mental Health | | | | Occupational Injuries | | | |
| --- | --- | --- | --- | --- | --- | --- | --- | --- | --- | --- | --- | --- |
|  |  | Interaction Term | LCI | UCI | Interaction Term | LCI | UCI |  | Interaction Term | LCI | UCI |  |
| Employment | Sex | 1.22 | 0.99 | 1.50 | 1.48 | 1.20 | 1.83 |  | 1.53 | 0.88 | 2.65 |  |
| Precariousness | Age | 1.01 | 1.00 | 1.01 | 1.01 | 1.00 | 1.01 |  | 1.00 | 0.99 | 1.00 |  |
|  | Education | 1.06 | 0.98 | 1.14 | 0.95 | 0.82 | 1.09 |  | 1.12 | 0.85 | 1.49 |  |
|  | Occupation | 0.60 | 0.50 | 0.72 | 0.86 | 0.76 | 0.98 |  | 0.64 | 0.38 | 1.09 |  |
|  |  |  |  |  |  |  |  |  |  |  |  |  |
| Insecurity | Sex | 0.65 | 0.41 | 1.06 | 0.63 | 0.44 | 0.90 |  | 1.75 | 0.50 | 6.08 |  |
|  | Age | 1.02 | 1.01 | 1.03 | 1.01 | 0.99 | 1.03 |  | 1.00 | 0.98 | 1.02 |  |
|  | Education | 0.71 | 0.38 | 1.33 | 0.84 | 0.58 | 1.22 |  | 0.50 | 0.24 | 1.02 |  |
|  | Occupation | 0.70 | 0.53 | 0.92 | 1.18 | 0.90 | 1.54 |  | 0.46 | 0.30 | 0.71 |  |
|  |  |  |  |  |  |  |  |  |  |  |  |  |
| Type of | Sex | 0.80 | 0.54 | 1.19 | 0.79 | 0.63 | 1.00 |  | 1.01 | 0.64 | 1.62 |  |
| Contract | Age | 1.03 | 1.01 | 1.06 | 1.02 | 1.00 | 1.04 |  | 1.00 | 0.94 | 1.06 |  |
|  | Education | 0.97 | 0.42 | 2.25 | 1.21 | 0.72 | 2.02 |  | 0.15 | 0.04 | 0.64 |  |
|  | Occupation | 0.53 | 0.33 | 0.85 | 0.73 | 0.47 | 1.14 |  | 0.54 | 0.20 | 1.51 |  |
|  |  |  |  |  |  |  |  |  |  |  |  |  |

Contrasts: Women vs. Men (reference group); age as a continuous variable; University vs Primary education (reference group); Non-Manuals vs Manuals (reference group).
